# Combining two repurposed drugs as a promising approach for Alzheimer's disease therapy

**DOI:** 10.1038/srep07608

**Published:** 2015-01-08

**Authors:** Ilya Chumakov, Serguei Nabirotchkin, Nathalie Cholet, Aude Milet, Aurélie Boucard, Damien Toulorge, Yannick Pereira, Esther Graudens, Sory Traoré, Julie Foucquier, Mickael Guedj, Emmanuel Vial, Noëlle Callizot, Rémy Steinschneider, Tangui Maurice, Viviane Bertrand, Catherine Scart-Grès, Rodolphe Hajj, Daniel Cohen

**Affiliations:** 1 Pharnext, 11 rue des Peupliers, 92130 Issy-Les-Moulineaux, France; 2 Neuro/Sys, 410 CD 60, 13120 Gardanne, France; 3 Neuronexperts, 51 bd Pierre Dramard, 13916 Marseille, France; 4 Université de Montpellier 2, 34095 Montpellier, France; Inserm, U710, 34095 Montpellier, France; EPHE, 75017 Paris, France; 5 Amylgen, 2196 bd de la Lironde, 34980 Montferrier-sur-Lez, France

## Abstract

Alzheimer disease (AD) represents a major medical problem where mono-therapeutic interventions demonstrated only a limited efficacy so far. We explored the possibility of developing a combinational therapy that might prevent the degradation of neuronal and endothelial structures in this disease. We argued that the distorted balance between excitatory (glutamate) and inhibitory (GABA/glycine) systems constitutes a therapeutic target for such intervention. We found that a combination of two approved drugs – acamprosate and baclofen – synergistically protected neurons and endothelial structures *in vitro* against amyloid-beta (A**β**) oligomers. The neuroprotective effects of these drugs were mediated by modulation of targets in GABA/glycinergic and glutamatergic pathways. *In vivo*, the combination alleviated cognitive deficits in the acute A**β**_25–35_ peptide injection model and in the mouse mutant APP transgenic model. Several patterns altered in AD were also synergistically normalised. Our results open up the possibility for a promising therapeutic approach for AD by combining repurposed drugs.

Alzheimer's disease (AD) is a lethal neurodegenerative disorder of older persons, affecting nearly 40 million patients worldwide. Clinically it is manifested by rapid progressive deterioration leading to the loss of independence. Mental capacities involving memory, language and everyday functioning are affected and this loss is often accompanied by psychosis. With the aging of world population and emerging of new effective treatments for other mortal diseases the importance of AD as an economic burden and limiting factor of life duration is progressively becoming more and more important. To date, all treatment options are marginally effective^1^ and preserve cognitive function only temporarily^2^. In AD, brain regions involved in cognitive functions show a series of abnormalities including progressive loss of neurons and synapses and signs of neuroinflammation, accompanied by neuronal degeneration^3^. In addition, the brain of AD patients often shows cerebrovascular defects such as abnormal permeability of the blood-brain barrier (BBB)^4^. Significant comorbidity between AD and cerebrovascular deregulation suggests that these defects are important features of AD^5^. Abnormal metabolism of amyloid precursor protein (APP) results in production of cytotoxic Aβ peptide oligomers and formation of extracellular amyloid plaques, histopathological hallmarks of AD. The attempts of therapeutic intervention at the level of processing and turnover of these peptides have failed so far^6^. The etiological factors leading to AD are not fully understood. They might act synergistically with amplifying feed-back loops^7^ implicating these peptides and other toxic factors, making the disease state resistant to mono-therapeutic interventions.

To affect the different elements that appear to contribute to this complex disorder, we are proposing here a new combinational approach. The systematic analysis of available genetic and functional data in the literature has drawn our particular attention to the disturbance in the balance between glutamate, GABA, and glycine systems for the development of pathological features prominent in AD that could play a role in degeneration of brain structures. Glutamate system in the brain is not only important for signal transmission and plasticity but also for the regulation of survival or induction of apoptosis of brain cells. This system is counter-balanced by inhibitory GABA and glycine signalling with normal brain functions assured by the subtle equilibrium between these inhibitory and excitatory (E/I) activities.

Deregulation of glutamate excitatory neuronal signalling has been proposed as a neurophysiological mechanism responsible for cognitive impairment in AD^8^. While GABAergic neurons are considered to be relatively preserved in AD patients^9^, a recent study has demonstrated a profound remodelling of molecular components of GABAergic neurotransmission^10^ with complex changes in the relative concentrations of its different molecular constituents, which by themselves could compromise the overall balance of E/I signalling.

We argued that the chronic presence of toxic Aβ oligomers could modify this balance, thereby impairing cognitive functions and enhancing the pro-apoptotic activity both in neuronal and endothelial cells. Thus, we have selected several candidate drugs, each potentially acting on multiple targets in these pathways. Since our ultimate goal has been to progress rapidly toward the AD treatment development, we focused on drugs that met two principal criteria: 1) used for a long time to treat other disorders^11^, and 2) having a good safety profile.

The first chosen drug is acamprosate calcium (ACP), an anti-craving agent that is thought to influence glutamatergic transmission and to affect the E/I balance distorted in ethanol addiction. ACP is a safe drug being used at exceptionally high doses. ACP decreases glutamate levels induced in human brain during ethanol withdrawal and reverses the resulting hyperexcitability^12^. Glutamate receptors are important both for the balance between cell death and survival^13^. Their function is substantially modified in AD^14^. Possible targets for ACP include the iono/metabotropic glutamate receptors^15^ but also inhibitory glycine gated ion channels^16^.

Furthermore, we thought that to be effective and to resist the possible feedback changes during chronic treatment, we should use a drug combination that acts also on the brain major inhibitory system: GABA. We therefore selected a second drug, (RS)-baclofen (BCL), used for the treatment of spasticity for several decades and which activates inhibitory metabotropic GABA_B_ receptors. BCL has rather favourable safety profile even when used at high doses and is the only agonist of GABA_B_ receptors that is authorised for human use. It should also be noticed that glutamate, GABA and glycine receptors targeted by ACP and BCL are also important for the function of the endothelial BBB^17^,^18^,^19^.

At this stage we decided to test ACP or BCL alone and in combination (ABC) in a battery of cellular and animal paradigms. No satisfactory model exists for AD^20^, but we concentrated our efforts on multiple observational endpoints that are mimicked in these models to provide a substantial rationale for the future testing in AD patients.

## Results

### ABC synergistically protects against Aβ toxicity in AD *in vitro* models

**Figure 1:**
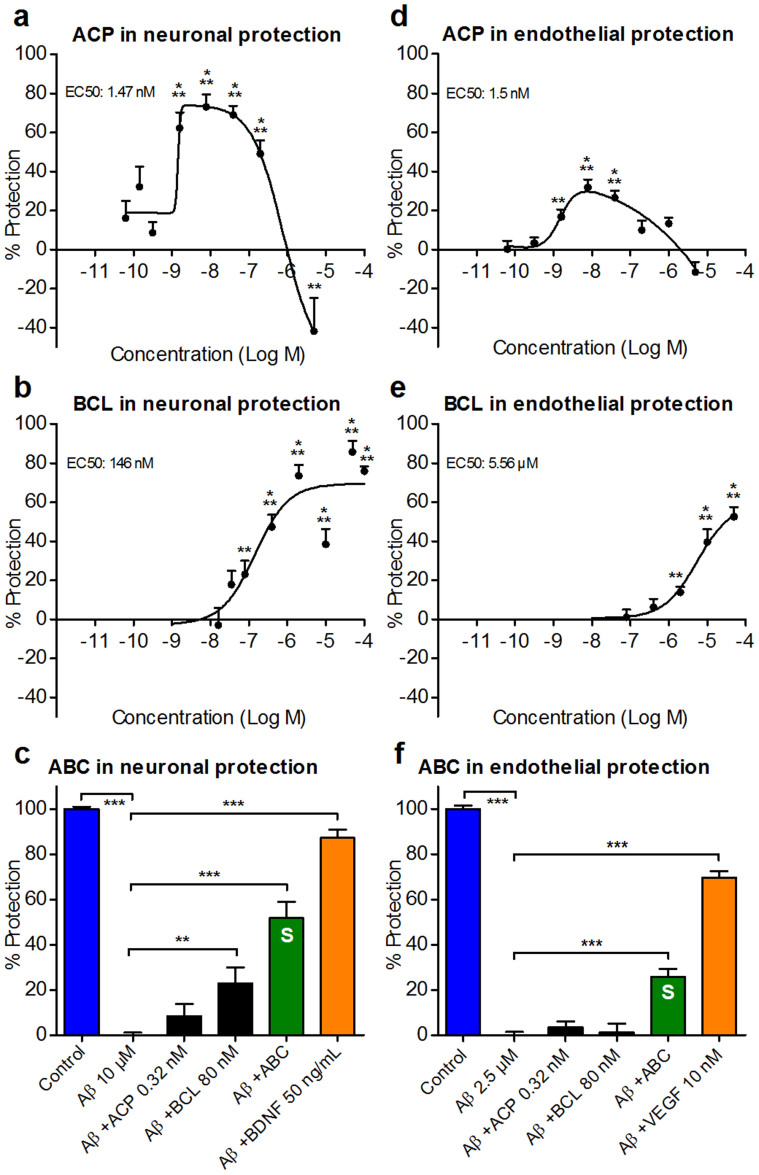
Combination of ACP and BCL acts synergistically to protect neurons and microvascular HBMEC tubules from the toxicity of Aβ_1–42_ oligomers. Normalised data for the difference between Aβ_1–42_ oligomer-treated and untreated cultures are presented. The dose-response curve of ACP in primary neurons and HBMEC (a,d) showed a bell-shaped protective aspect in both assays, while that of BCL was sigmoid (b,e). At sub-active concentrations of individual drugs, ABC was active with a synergistic effect on neuronal (c) (*n* = 7) and HBMEC tubular (f) (*n* = 3) protection. BDNF (c) and VEGF (f) were used as controls. All values are mean ± s.e.m. **P < 0.01, ***P < 0.001 versus Aβ; ANOVA with Dunnett's test. S: Synergy.

To test the protective potential of ABC against the effects of toxic soluble oligomeric Aβ_1–42_ peptides, we used two models: a primary culture of rat cortical neurons^21^, and a primary culture of human brain-derived microvascular endothelial cells (HBMEC) that form a tubular network in matrigel^22^. We used the same batch of Aβ_1–42_ peptides described by us previously^21^. This batch, extensively studied by several techniques contained low and high molecular weight Aβ_1–42_ oligomers as assessed by Western blot analyses using the 6E10 antibody. We verified that only preparations containing Aβ_1–42_ oligomers were able to induce toxicity in neuronal cultures, while initial peptide preparation that did not contain oligomers was not toxic^21^, excluding a nonspecific effect of contaminating agents. This allowed us to use non-treated cells as controls without the need for using scrambled peptides as negative controls. At low doses, ACP (Fig. 1a,d) and BCL (Fig. 1b,e) alone protected neurons as well as the microvascular network against Aβ_1–42_ toxicity (Supplementary Table 1). To test for possible additive or synergistic interactions between the two drugs, sub-active doses (doses only marginally active in both assays) were used (Supplementary Fig. 1a,b). We found significantly greater neuronal and vascular cell protection from Aβ_1–42_ toxicity with ABC compared to the effect of each drug individually (Fig. 1c,f). This positive interaction between ACP and BCL was confirmed by calculation of Combination Indexes and isobolograms (Supplementary Fig. 1c) for a range of ABC doses and ratios (Supplementary Fig. 1d). Thus, we demonstrated that the effect of the two drugs in combination was not simply additive but synergistic, which highlighted the advantage of their combination.

### Molecular features of AD are improved by ABC *in vitro*

**Figure 2:**
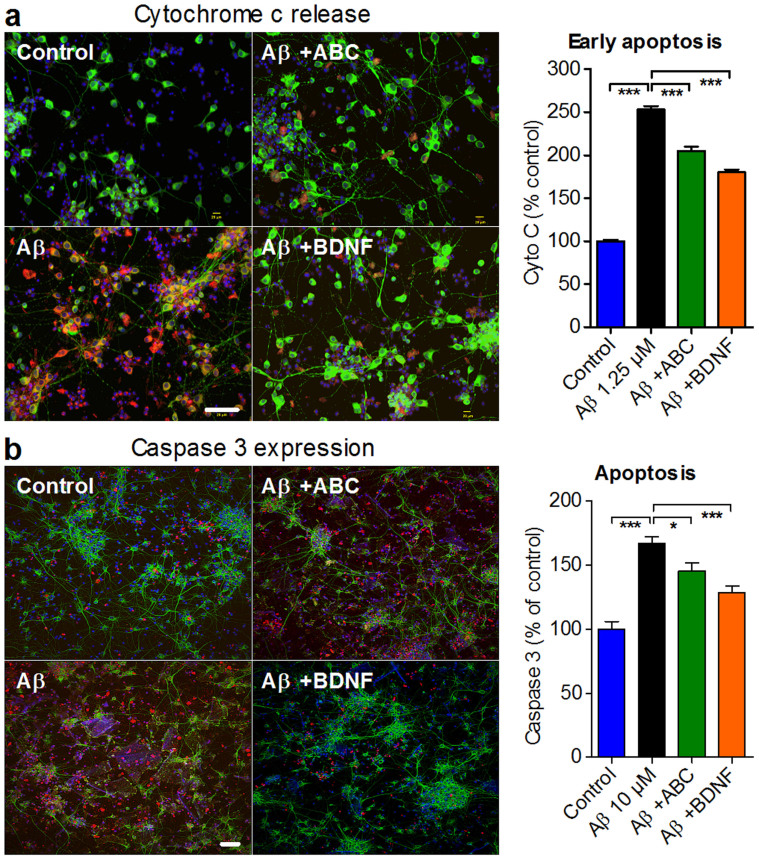
ABC action to reduce Aβ_1–42_-induced cell death is mediated by preventing apoptosis. Positive effect of ABC on apoptosis assessed by Cyto c (a) and caspase 3 (b) staining. Neurons with Cyto c or caspase 3 (in red) overlapping with MAP2 (in green) were counted and results were expressed as percentage of untreated control. Scale bars: 60 μm. Values are mean ± s.e.m. *P < 0.05, ***P < 0.001 versus Aβ; ANOVA with Dunnett's test. ABC was composed of 0.32 nM ACP and 80 nM BCL. BDNF was used as positive control (50 ng mL^−1^). MAP2: Microtubule-associated protein 2. Blue: Hoechst.

**Figure 3:**
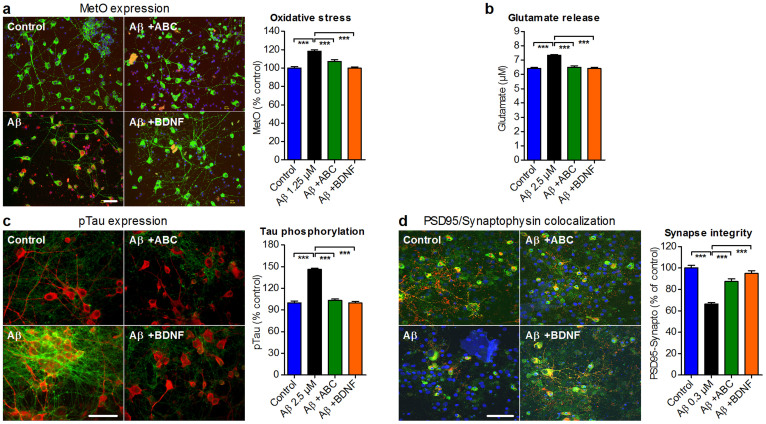
ABC protects Aβ_1–42_-intoxicated neuronal cells from apoptosis by normalising several induced alterations. (a) ABC reduced oxidative stress as assessed by MetO staining. Neurons with MetO (in red) overlapping with MAP2 (in green) were counted and results were expressed as percentage of untreated control. (b) Normalisation by ABC of cell-released glutamate quantified in the medium of Aβ-intoxicated neurons. (c) Preservation by ABC of phosphorylated tau protein (pTau^Ser212/Thr214^, green) quantified in Aβ-intoxicated neurons (red). (d) Synaptic integrity was preserved following ABC treatment, as assessed by the co-localisation of pre-synaptic synaptophysin (red) and post-synaptic PSD95 (green) proteins. Scale bars: 60 μm. Values are mean ± s.e.m. ***P < 0.001 versus Aβ; ANOVA with Dunnett's test. ABC was composed of 0.32 nM ACP and 80 nM BCL. BDNF was used as positive control (50 ng mL^−1^). MetO: Oxidised methionines; PSD95: Post-synaptic density protein 95. Blue: Hoechst.

Mitochondrial dysfunction is believed to play a critical role in AD pathology^23^ triggering apoptosis. We sought to check whether neuronal protection by ABC mechanistically involves the improvement of this dysfunction. We found that Aβ_1–42_ oligomers increased the release of mitochondrial cytochrome c into the cytoplasm (Fig. 2a), which was accompanied by caspase 3 activation (Fig. 2b) leading to apoptosis, but ABC was able to protect intoxicated neuronal cells from this apoptotic activation (Fig. 2). Oxidative stress is associated to mitochondrial dysfunction and plays a role in the development of AD pathology^24^. We assessed oxidative stress by measuring the levels of methionine residues oxidation (MetO) in neurons and showed that ABC significantly protected neuronal cells against such stress (Fig. 3a). The presence of toxic Aβ species in the brain of AD is believed to bring excessive glutamate accumulation^25^ affecting synapse integrity^8^ and triggering loss of neuronal cells. We demonstrated *in vitro* that ABC was able to diminish this Aβ_1–42_-induced toxic glutamate increase (Fig. 3b). Tau protein hyperphosphorylation that brings its abnormal cellular distribution and aggregation into neurofilamentary tangles^26^ is another important molecular feature of AD disease. It is believed to be an additional toxic factor leading to brain degradation in AD patients^27^. We found, that ABC was able to prevent Tau hyperphosphorylation induced by Aβ_1–42_ in cellular cultures (Fig. 3c).

Synaptic loss is prominent in affected brains and is largely responsible for the deterioration of the cognitive function. In neuronal cultures, Aβ_1–42_ oligomers induced a substantial synaptic loss, but ABC prevented this loss (Fig. 3d) further explaining the observed neuroprotection.

In all *in vitro* experiments above, we used BDNF exclusively as a positive control for quality control purposes.

### Molecular targets important for ACP and BCL mechanism of action in neuroprotection against Aβ toxicity

**Figure 4:**
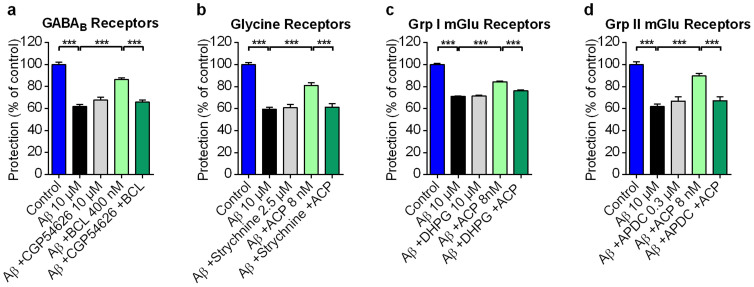
ACP and BCL act through GABA_B_, Glycine and metabotropic glutamatergic receptor signalling. GABA_B_R CGP54626 antagonist (a), Glycine receptor Strychnine antagonist (b), mGluR1/5R DHPG agonist (c) and mGluR2/3R (2R,4R)-APDC agonist (d) all blocked BCL and ACP protection of rat primary neuronal cells validating the action of these drugs on these pathways. Values are mean ± s.e.m. ***P < 0.001 versus Aβ, BCL or ACP; ANOVA with Dunnett's test. mGlu1/5R and mGlu2/3R: metabotropic glutamate group I and II receptors respectively.

In order to confirm our hypothesis that elements in the excitatory glutamatergic system as well as the regulation through GABA and glycine receptor systems are playing a role in the neuroprotection against Aβ toxicity, we tested the relevance of some plausible targets in these pathways through a pharmacological approach. CGP54626, orthosteric antagonist of GABA_B_ receptors, was able at nontoxic doses to block the neuroprotective effect of racemic BCL (Fig. 4a), which confirms the importance of GABA_B_ receptor activation for the neuroprotective action of BCL. When strychnine, an antagonist of inhibitory glycine-gated channels, was used with ACP, it was also able to reverse the neuroprotective effect (Fig. 4b), pointing to the mediation of neuroprotection induced by ACP through its agonistic effect on ionotropic glycine receptors. On the other hand, similar experiments with the agonists DHPG and APDC, acting on group I and II metabotropic glutamate receptors respectively, clearly demonstrated the importance of the antagonistic effect of ACP on these molecular targets in excitatory glutamatergic transmission (Fig. 4c,d). Taken together, these results confirm the activity of our drugs on multiple receptors implicated in the GABAergic/glycinergic and glutamatergic signalling systems.

### ABC alleviates cognitive deficits in AD *in vivo* models

To explore the effects of ABC in a functional context, we used a well-known model of intracerebroventricular injection of Aβ_25–35_ oligomers in mice to mimic both the cognitive impairment and the associated cellular degeneration observed in AD^28^,^29^. Aβ_25–35_ oligomerises in a similar manner to the larger Aβ_1–42_ peptide, forming β-sheet amyloid-like short fibrils and induces toxicity with a similar pattern^30^. Oligomeric preparations used in our study have been extensively characterised by us for the presence of toxic oligomers^29^,^30^, and were confirmed to produce AD-like symptoms *in vivo*^30^. Aβ_25–35_, a fragment present in Aβ_1–42_, was used for *in vivo* intracerebroventricular intoxications because it is more diffusible due to its smaller size. It was shown to penetrate in brain structures and produce a lasting toxic effect of over 6 weeks^30^ only after one injection, unlike Aβ_1–42_ with its toxic effect on memory recovering after 10 days^31^. Aβ_25–35_ has been shown to have multiple biochemical effects on intracellular compartments similar to what is observed in AD patients^32^. Moreover, production of endogenous Aβ_1–42_ peptides could be easily and specifically monitored in Aβ_25–35_-injected mice with the help of selective antibodies for the Aβ_1–42_ forms. The same batches of oligomerised Aβ_25–35_ peptides as well as control, nontoxic Sc.Aβ peptides with the same amino acid composition were used in our study as described by Zussy *et al*^30^. ACP and BCL dose selection was based on pharmacokinetics studies and modelling for the translation of drug concentrations effective *in vitro* to animal doses. For all tests below, treatment durations are detailed in Supplementary Table 2.

**Figure 5:**
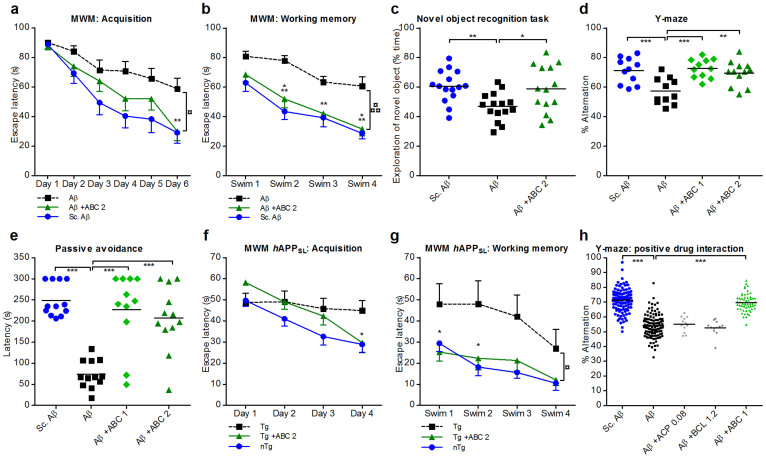
ABC alleviates cognitive deficits in AD mouse models. Intracerebroventricularly Aβ_25–35_-injected Swiss male mice compared to Sc.Aβ controls: Positive effect of ABC in the Morris Water Maze (MWM) acquisition task (a) and working memory tests (b) (*n* = 12). Positive effect of ABC in NOR task (c) (*n* = 15), and in representative Y-maze (d) and passive avoidance (e) experiments (*n* = 12). Locomotor activities of Sc.Aβ, Aβ, and treated mice were unchanged in Y-maze and NOR tests (Supplementary Fig. 5). Transgenic *h*APP_SL_ C57BL/6 male mice: Positive effect of ABC in MWM acquisition (f) and working memory tests (g) (*n* = 12). (h) ABC superior activity over its single drugs (combined analysis (see Supplementary Fig. 3)). Values are mean ± s.e.m. *P < 0.05, **P < 0.01, ***P < 0.001 versus Aβ (c–e,h) (ANOVA with Dunnett's test) or ABC versus Aβ (a,b) and ABC versus Tg (f,g) (mixed ANOVA with Dunnett's test). ^¤¤^*P* < 0.01, ^¤¤¤^*P* < 0.001 global ABC versus global Aβ (a,b), or global ABC versus global Tg (f,g) (mixed ANOVA with Dunnett's test). ABC 1: ACP 0.08 mg Kg^−1^ + BCL 1.2 mg Kg^−1^; ABC 2: ACP 0.2 mg Kg^−1^ + BCL 3 mg Kg^−1^. 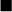: Aβ (a,b) or Tg (f,g); 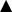: Aβ (a,b) or Tg (f,g) + ABC 2; •: Sc.Aβ (a,b) or nTg (f,g). Tg: transgenic; nTg: nontransgenic. Sc.: scrambled.

The effect of the combination of ACP and BCL at the selected doses was first assayed in the Morris Water Maze (MWM) test, which explores hippocampal-dependent spatial memory^33^, a form of memory affected early in AD. ABC showed a significant protection against cognitive impairment due to Aβ_25–35_ injury in the acquisition of long-term memory (Fig. 5a), as well as in the short-term memory test (Fig. 5b). We also used the novel object recognition (NOR), another memory task affected by hippocampal and cortical-mediated alterations^34^. Aβ_25–35_-injected mice demonstrated deficits in NOR, while upon ABC treatment, the animals substantially and significantly recovered the ability to recognise novel objects (Fig. 5c). ABC was also able to restore this capacity in an analogous rat model assayed in another laboratory (Supplementary Fig. 2). Further, we evaluated ABC action in spontaneous alternation Y-maze test, which is partly linked to the septo-hippocampal system implicated in learning and spatial working memory^35^. In this test, ABC also substantially and significantly reversed alternation deficit induced by Aβ_25–35_ (Fig. 5d). Finally, we tested ABC in a passive avoidance test used to assess hippocampal and amygdala dependent aversive memory in animals^36^. ABC fully restored memory deficits induced by Aβ_25–35_ in this assay (Fig. 5e).

A cognitive decline is also observed in mouse transgenic models of AD^37^ that possibly mimics some aspects of the prodromic phases of human disease. At the age of 8 months, when *h*APP_SL_ transgenic mice cognition was already impaired, we treated animals for 3–4 weeks with ABC and found a substantial improvement of cognitive deficits assessed by MWM acquisition and working memory tests (Fig. 5f,g).

Since ABC showed synergism between drugs *in vitro*, we then tested ACP, BCL and ABC in the Y-maze test in a dose-dependent fashion in the acute peptide injection model. We observed a limited activity for ACP or BCL alone for certain doses, but the ABC effect was dose-related and consistently and significantly stronger (Supplementary Fig. 3), demonstrating a formal synergistic interaction between these two drugs *in vivo* as well (Fig. 5h). We found that ABC did not affect the basal cognition of age-matched naive mice and mice injected with control Aβ_25–35_ scrambled peptide (Supplementary Fig. 4), showing the limitation of action of ABC treatment to the pathological condition induced in this model by Aβ_25–35_.

### Mechanistic aspects of ABC regarding its action on the functional preservation *in vivo*

**Figure 6:**
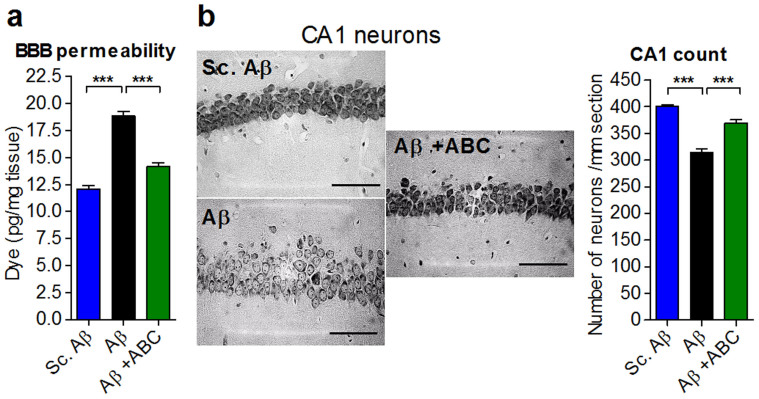
ABC preserves phenotypic alterations occurring *in vivo* in Aβ_25–35_-injected animals. ABC protected the BBB from leakage as assessed by Evans Blue dye in brain after systemic injection (a) (*n* = 12), and preserved hippocampal CA1 pyramidal cells (b) (*n* = 12). (b) Representative images of Cresyl violet-stained hippocampal CA1 neurons before and after ABC treatment. Hippocampal CA1 pyramidal cells were counted from 8 slices per animal and 6 mice per group and results were expressed as number of neurons per mm. ABC was composed of ACP 0.2 mg Kg^−1^ and BCL 3 mg Kg^−1^. Values are mean ± s.e.m. ***P < 0.001 versus Aβ; ANOVA with Dunnett's test. Sc.: scrambled.

**Figure 7:**
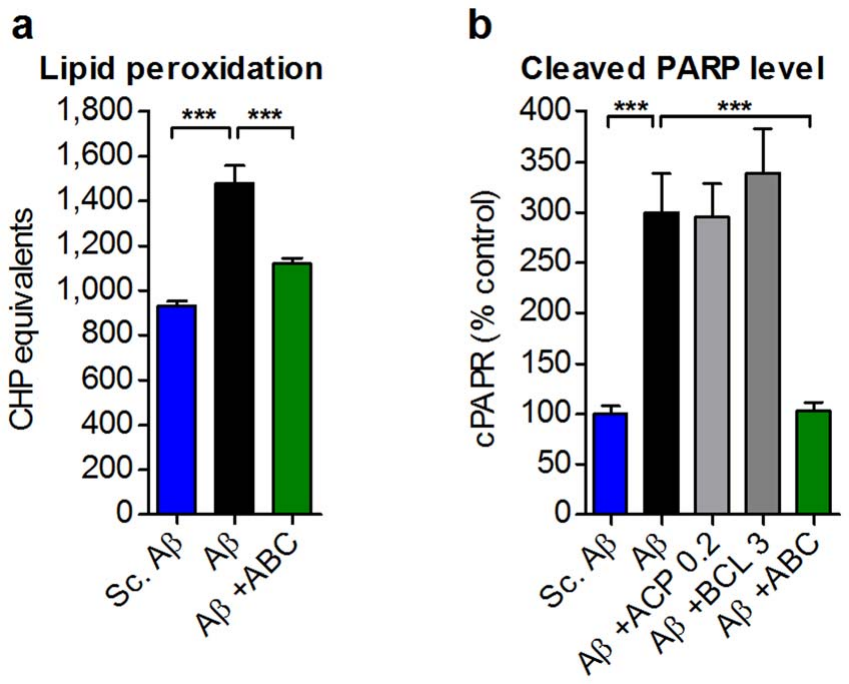
ABC protects from oxidative stress and apoptosis *in vivo* in Aβ_25–35_-injected animals. (a) Decrease in oxidative stress assessed by lipid peroxidation in mice hippocampi following ABC treatment (*n* = 6). (b) cPARP levels were normalised by ABC in mice hippocampi (*n* = 6). A higher efficacy was observed for ABC with respect to its single drugs. ABC was composed of ACP 0.2 mg Kg^−1^ and BCL 3 mg Kg^−1^. Values are mean ± s.e.m. ***P < 0.001 versus Aβ; ANOVA with Dunnett's test. CHP: cumene hydroperoxyde; Sc.: scrambled.

ABC proved its potential to alleviate Aβ_25–35_-induced cognitive deficits *in vivo* in several models and memory paradigms. To understand what brought the beneficial effect of ABC on cognition, we addressed several changes relevant to the human disease. First, we asked whether brain integrity is preserved by ABC and looked for BBB alterations after acute injection of Aβ_25–35_. Following ABC treatment, BBB was preserved in injured mice as assessed by systemic injection of Evans Blue dye and quantification in brain (Fig. 6a). Hippocampus is one of the main regions of the brain affected at the early stages of AD and it is severely compromised in the Aβ_25–35_ acute injection model^28^,^29^. When Aβ_25–35_-injured animals were treated with ABC, CA1 neurons were substantially and significantly preserved (Fig. 6b). Since CA1 loss in AD could be a consequence of oxidative stress and apoptosis^38^, we also tested the ability of ABC to normalise these alterations induced by Aβ_25–35_ *in vivo*. We found that ABC prevented lipid peroxidation (Fig. 7a), a marker for such stress, and protected neuronal cells from apoptosis as assessed by cleaved poly-ADP-ribose polymerase level (cPARP)^39^ (Fig. 7b).

**Figure 8:**
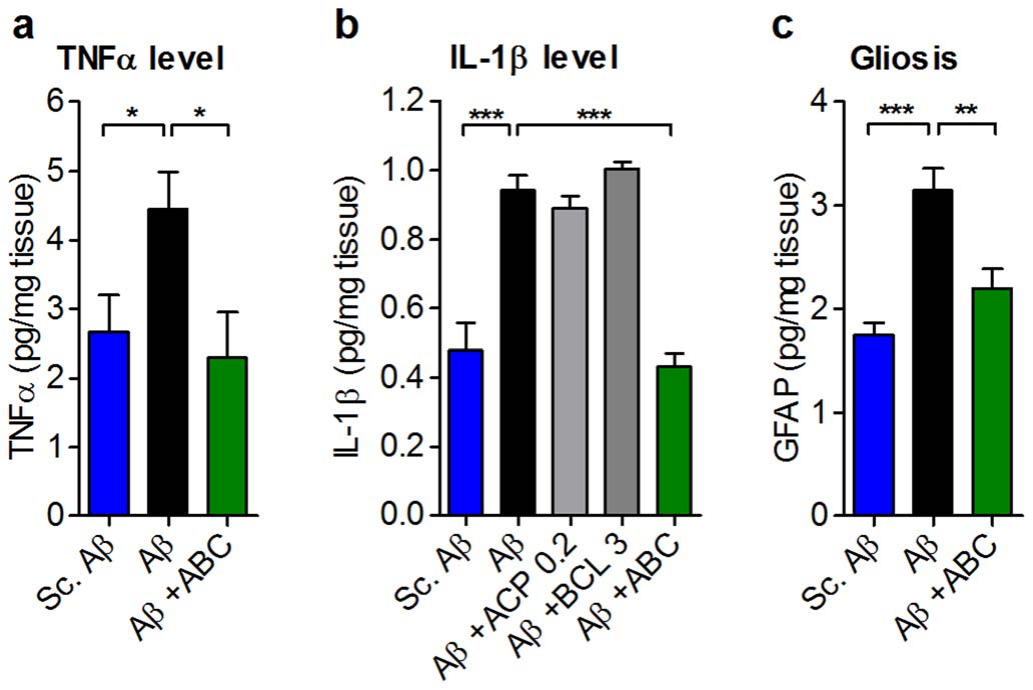
ABC normalises inflammation and gliosis *in vivo* in Aβ_25–35_-injected animals. Normalisation by ABC of TNFα (a) and IL-1β (b) protein levels in mice hippocampi (*n* = 6). A higher efficacy was observed for ABC with respect to single drugs. (c) Gliosis, assessed by GFAP protein quantification, was also normalised in mice hippocampi following ABC treatment (*n* = 6). ABC was composed of ACP 0.2 mg Kg^−1^ and BCL 3 mg Kg^−1^. Values are mean ± s.e.m. *P < 0.05, **P < 0.01, ***P < 0.001 versus Aβ; ANOVA with Dunnett's test (except in (a), *t*-test). GFAP: glial acidic fibrillary protein; Sc.: scrambled.

Another feature of AD is neuroinflammation^40^ accompanied by elevated levels of pro-inflammatory lymphokines released by activated microglia. To examine the effect of ABC on this inflammatory process, we quantified the proinflammatory cytokines TNFα and IL-1β and found their levels to be reduced to normal upon ABC treatment (Fig. 8a,b). One of the described consequences of brain degeneration and neuroinflammation in AD is glial scarring^41^. When Aβ_25–35_-injured mice were treated with ABC, we found that gliosis was normalised (Fig. 8c).

**Figure 9:**
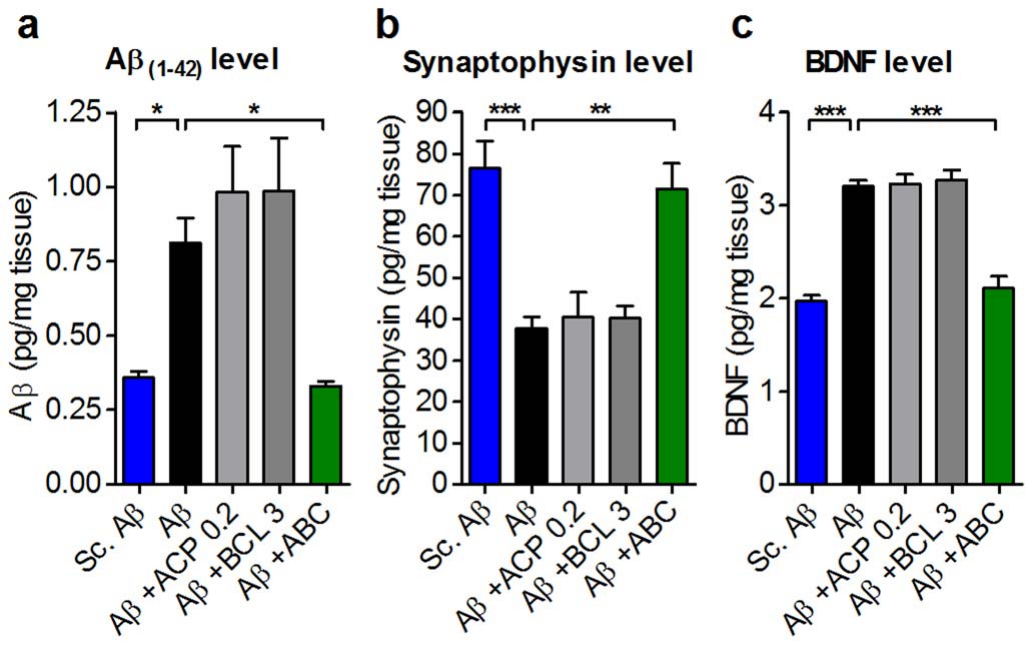
ABC protects from Aβ_1–42_ accumulation and synaptic loss, and from consequent downstream BDNF deregulation *in vivo*. ABC protected from Aβ_1–42_ endogenous secretion in mice hippocampi (a) (*n* = 6) preserving synapse integrity assessed by synaptophysin protein quantification (b) (*n* = 6). (c) BDNF protein level was normalised in mice hippocampi following ABC treatment (*n* = 6). A higher efficacy was observed for ABC with respect to single drugs. ABC was composed of ACP 0.2 mg Kg^−1^ and BCL 3 mg Kg^−1^. Values are mean ± s.e.m. *P < 0.05, **P < 0.01, ***P < 0.001 versus Aβ; ANOVA with Dunnett's test. Sc.: scrambled.

Accumulation of endogenous oligomeric Aβ_1–42_ is one of the toxic factors responsible for symptoms observed in AD^42^. Its toxicity could mediate many of the alterations described above or be amplified by them. When mice were exposed to exogenous Aβ_25–35_, we observed a substantial and significant increase in endogenous Aβ_1–42_, confirming the previously reported findings^43^ and suggesting a positive feed-back loop in the formation of toxic Aβ species. This overproduction was fully and significantly normalised by ABC treatment (Fig. 9a). As synaptic alterations observed in AD could result from the accumulation of toxic Aβ oligomers, we tested ABC for its ability to preserve synaptic loss. When synaptophysin marker was used to this goal we observed its substantial and significant normalisation (Fig. 9b). Another important consequence of Aβ_1–42_ accumulation is dysregulation in the amount of brain-derived neurotrophic factor (BDNF). This factor is thought to undergo a compensatory increase under conditions of Aβ-associated neurotoxicity^44^, that is supposed to aid in neuronal protection. We confirmed that the level of BDNF was significantly increased in Aβ_25–35_-injured mice, but it was maintained at normal levels by ABC treatment (Fig. 9c) that probably acted upstream of the BDNF compensatory induction.

When we compared the effect of single drugs to that of ABC for several biochemical features, we observed the same strong positive interaction between ACP and BCL that we demonstrated before in the Y-maze memory paradigm (Supplementary Fig. 3 and Fig. 5h). It was evident from synergistic effects on apoptosis, inflammation (IL-1β), endogenous Aβ_1–42_ accumulation, synapse integrity and BDNF levels (Figs 6 to 9).

## Discussion

The multifactor nature of AD might be one of the reasons why past mono-therapeutic attempts were not particularly effective. The underlying idea of our approach was to explore the possibility of developing combination therapy acting downstream of the formation of toxic Aβ species that might robustly prevent the degradation of both neuronal and endothelial cellular structures affected in this disease. We therefore considered that such treatment could potentially have a disease modifying effect. We were guided by the fact that many signalling pathways are pleiotropic^45^, which opens up the possibility of designing new treatments based on approved drugs acting on them. In combination, such drugs might lead to an enhanced therapeutic effect at a lower dosage^46^, further decreasing the risk of potential undesirable events.

Our analysis of regulators acting on interconnected signalling pathways led to two well-known drugs with rather favourable safety profile: BCL and ACP. Some of their targets are well documented, while the action on others could be indirect and dose specific, especially in the case of ACP usually used at extremely high doses. Metabotropic GABA_B_ receptors play a fundamental role in controlling the degree of neuronal activity. At glutamatergic synapses, presynaptic GABA_B_ heteroreceptors can decrease the release of glutamate from the presynaptic membrane by inhibiting Ca^2+^ channels, while postsynaptic GABA_B_ receptors attenuate excitatory currents by activating Kir3-type K^+^ channels^47^. Therefore, we suppose that activation of GABA_B_ receptors by agonistic action of BCL can restore the disequilibrium induced by the dysregulation in glutamatergic signalling that is provoked by neurotoxic forms of Aβ. Additionally, activation of GABA_B_ receptors by BCL could attenuate, via IGF-1 receptor, the pro-apoptotic signalling specifically engaged by neurotoxic Aβ protein^48^. Inhibitory receptors like GABA_B_ are also present in endothelial cells, suggesting that the balance between excitatory and inhibitory neurotransmitters could play an important role not only in the regulation of brain functions, but also in the functioning of the vascular system^49^,^50^. Therefore, activation of inhibitory GABA_B_ receptors can be recognised as a promising therapeutic strategy for decreasing excessive activity of glutamatergic signalling and restoring E/I balance. In our study, we were able to confirm the strong neuroprotective effect of BCL *in vitro*. This effect disappeared in the presence of orthosteric antagonist of GABA_B_ receptors, experimentally confirming the on-target agonistic action of BCL in this model. The second drug, ACP (calcium salt of acetyl homotaurine), was first approved in 1989 and since then has been used to treat millions of alcohol-dependent patients worldwide^51^. It was shown to reduce relapse in alcohol-dependent patients through restoration of the balance between excitatory and inhibitory transmission affected by chronic alcohol use. ACP could decrease glutamate level in human brains, induced by hyperexcitability during episodes of ethanol withdrawal^12^. It is supposed to attenuate hyper-glutamatergic states that occur during abstinence, via inhibitory effects on glutamate receptors and potentiation of inhibitory neuronal signalling. However, its precise mechanism of action in addiction treatment remains largely unknown. Moreover, it has been suggested that at least a part of its effects can be due to the presence of calcium ions^52^. In our neuroprotection experiments, ACP has been effective in a fraction of nanomolar concentrations, excluding its effect due to the presence of calcium ions. Moreover, we demonstrated experimentally that ACP neuroprotective effect was specifically mediated by N-acetyl homotaurine (ACP) and not by calcium ions contained in its calcium salt (Supplementary Fig. 6).

Calcium ACP was shown to be a weak modulator of NMDA receptors^53^,^54^, but its action is probably not direct. Since sodium ACP did not compete with NMDA for glutamate binding sites but displayed total competition with trans-ACPD^55^, a nonspecific agonist for group I and II metabotropic glutamate receptors (mGluRs), we decided instead to evaluate a role of metabotropic glutamate receptors for neuroprotective effects observed by us. The role of these receptors in the pathogenesis of AD is not yet fully established, and published observations are limited. mGluR2, belonging to group II glutamate receptors, was found to be overexpressed in the hippocampus of AD patients^56^. Caraci *et al.* demonstrated that a positive allosteric modulator of group II mGluR2 aggravated Aβ-induced neurotoxicity^57^, while knockout of group I mGluR5, considered as a co-receptor for Aβ^58^, attenuates cognitive impairment in a mouse model of AD^59^. APP has been shown to be rapidly translated in the synapses in response to DHPG, agonist of group I mGluRs^60^, and such activation stimulates the release of Aβ_1–42_ in cortical synaptoneurosomes in a mouse model of AD, while stimulation of group II mGluRs triggers production and release of both Aβ_1–42_ and Aβ_1–40_^61^. By a pharmacological approach with the help of two broadly specific agonists, we demonstrated experimentally that the neuroprotective action of ACP is mediated by its antagonistic effect on both group I and group II mGluRs.

In the ethanol addiction model, ACP has been shown to act in the nucleus accumbens through agonism on glycine receptors (GlyRs)^16^. These inhibitory channels are expressed throughout all regions of the hippocampus^62^. At the synapses, they are co-localised with GABA_A_ receptors but mostly are found in extrasynaptic sites. In our experiments, we demonstrated that neuroprotection by ACP is also mediated by its agonistic action on GlyR channels. We hypothesise that activation of GlyRs by ACP might be an important factor for the attenuating excessive/aberrant neuron excitability, such as deleterious over-activation of extrasynaptic NMDA receptors by Aβ^8^. These experiments suggested that, for the neuroprotective action of ACP against Aβ_1–42_ toxicity, its antagonistic signalling through both Group I and group II metabotropic glutamate receptors as well as agonistic activation of GlyR channels is required, while BCL action is mediated by activation of GABA_B_ receptors. Yet, our data is not conclusive at this stage but we suppose that neuroprotective effects of ABC could be mediated by a serial signalling cascade involving all these receptors. Future work is needed to further characterise the mode of action of ABC in this regard as well as its specificity on our studied targets.

While no comprehensive model for Alzheimer's disease exists^20^, representing the major limitation for the discovery of effective drugs, multiple observational endpoints relevant to the human disease were improved by ABC in several cellular and animal paradigms modelling different aspects of AD. *In vitro*, ACP and BCL proved to be cytoprotective both for neurons as well as for vascular cells with strong synergistic effect and at relatively low doses. The exact mechanisms explaining such action are only emerging, but we found that neuroprotection could be due to the down-regulation of glutamate release, tau phosphorylation and diminished oxidative stress, and that it is mediated by an antagonistic action on metabotropic receptors of classes I/II and an agonistic action on GlyR in the case of ACP, and activation of GABA_B_R in the case of BCL.

The acute model of intracerebroventricular injection of Aβ peptide into healthy rodents mimics main features of AD-type dementia including neuronal degeneration, cognitive deficits accompanied by hippocampal dysfunction^28^ and BBB impairment^63^. Since cognitive function and memory do not have a complete analogy between rodents and humans, we tested the influence of our drug combination in several memory paradigms. We observed a strong and synergistic improvement of cognition accompanied by substantial neuronal protection and BBB preservation. The important biochemical landmarks (Aβ_1–42_, oxidative stress, inflammation) of AD were significantly diminished by the drug combination. The effect was not due to a simple memory enhancement since in animals injected with nonspecific scrambled Aβ peptide as well as in normal animals cognition was not affected by ABC. In addition, ABC proved to be effective when tested in the transgenic mouse model at a stage when cognitive functions were already altered. Together, all these results open the possibility that ABC could exert a disease modifying effect. Already, we demonstrated that several patterns altered in AD, such as glutamate accumulation, tau hyperphosphorylation, endogenous Aβ oligomers and synaptic loss were synergistically normalised by ABC.

Our results demonstrate the value of 1) the combinational approach and 2) using for this purpose previously existing drugs in new combinations^11^. Many new drugs effective in preclinical models failed in human trials due to their toxic effects, while ACP and BCL have been used for a long time to treat patient populations and demonstrated very limited degree of side effects. The synergistic potency of the combination could allow further reduction of therapeutic doses and thus lower the risk for side effects. Indeed, we consistently observed a positive effect at low doses as we progressed from *in vitro* studies to animal models. Over 200 compounds have been shown to affect favourably AD-like pathology in experimental models^64^. However, to our knowledge, none of them have shown a simultaneous *in vitro* and *in vivo* improvement of the number of diverse biological AD related features as our combination. It protected both neuronal and vascular brain cells from Aβ insults, preserved synapses and reduced excessive tau protein phosphorylation, while at the same time reduced soluble endogenously produced Aβ peptides, diminished the signs of neuroinflammation and gliosis and alleviated cognitive deficits in different memory paradigms in AD animal models. We consider that the integrity of our data suggest that ABC might be a valuable disease-modifying treatment for AD, acting through a new mechanism implicating multiple molecular targets. If confirmed by powered clinical testing, low-dose ABC could serve a basis for a new conceptual approach for Alzheimer's disease therapy and also might help to understand the underlying mechanisms of this devastating dementia.

## Methods

### Animals

All animal procedures were carried out in strict adherence to the European Community Council Directive of September 22, 2010 (2010/63/UE). All experiments and protocols were authorised and approved by the French Ministry of Research, as well as by Animal Welfare Committees of Amylgen, Biotrial, QPS and Neuronexperts. All efforts were made to minimise the number of animals used. Male Swiss mice (Janvier, Saint Berthevin, France) aged 4–6 weeks and weighing 25–32 ± 2 g, and male Sprague Dawley rats (Janvier) aged 7–8 weeks weighing 220–300 g were used for Aβ_25–35_ intoxications. For transgenic mice experiments, male mice overexpressing the 751 amino acid form of human APP (*h*APP) with London (V717I) and Swedish (KM670/671NL) mutations (*h*APP_SL_) under the control of the murine Thy-1 promoter^65^ were bred at QPS (Austria), and C57BL/6 age and gender matched transgenic littermates served as controls (nTg). Animals were housed in plastic cages with free access to food and water, except during behavioural experiments, and kept in a regulated environment (23 ± 1°C, 50–60% humidity) under a 12 h light/dark cycle. For *in vitro* experiments, female Wistar rats (Janvier) on gestational day 15 were killed by cervical dislocation and foetuses were removed from the uterus.

### Drugs and treatments

Aβ_1–42_ peptide (Bachem, Reference H1368, Batch 1010533) was prepared in serum-free culture medium (0.1% DMSO) at 40 μM and was slowly shaken at +37°C for 3 days in the dark. After 3 days, Aβ_1–42_ oligomers were used on primary cortical neurons at specified concentrations and for indicated incubation times depending on the assay (Supplementary Table 1). It is worthy of note that, for *in vitro* and *in vivo* experiments, when a new batch was selected, Aβ preparations were always verified for the presence of Aβ oligomers. In dose-response studies *in vitro*, these preparations were tested using the appropriate reference compounds such as BDNF and β-estradiol (which rescue Aβ toxic effect) to check for reproducibility between studies. In addition, we note that our Aβ_1–42_ oligomeric preparation was performed in media containing phenol red at concentrations (i.e. 44 μM) that did not affect oligomeric formation as assessed by Western blot analyses attesting for the presence of these toxic oligomers^21^ that were active in our experimental settings. It was also described elsewhere that high concentrations of phenol red should be used over several days to inhibit efficiently Aβ_42_ oligomerisation^66^.

The amyloid-β[25–35] peptide (Aβ_25–35_) and scrambled control Aβ_25–35_ (Sc.Aβ) peptide were purchased from Genepep (Saint-Jean-de-Védas, France) for mice and from Bachem (Germany) for rat experiments. Peptides were solubilised in distilled water at a concentration of 3 mg mL^−1^ (mice) or 2 mg mL^−1^ (rats) and stored at −20°C until use. Before injection, peptides were incubated at +37°C for 4 days, allowing Aβ_25–35_ to form oligomers. A quality control analysis for Aβ_25–35_ aggregation was systematically performed by light scattering analysis before each injection of Aβ_25–35_ peptides. 9 nmol per mouse or 15 nmol per rat of Aβ_25–35_ peptide or Sc.Aβ were administered once icv in a final volume of 3 μL per mouse or 8 μL per rat^29^.

(RS)-Baclofen and acamprosate calcium were provided by Sigma Aldrich. Acamprosate sodium was synthesised by NovAlix (Illkirch, France). Drugs were solubilised in distilled water and prepared before each administration. For *in vitro* studies, ACP, BCL or ABC dissolved in 0.1% DMSO were added 1 h or 48 h before Aβ_1–42_ intoxication and maintained until the end of Aβ intoxication (Supplementary Table 1). For mechanism of action studies, non-toxic concentrations of CGP54626 (10 μM, GABA_B_R antagonist, Tocris Biosciences), Strychnine (2.5 μM, Glycine receptor antagonist, Tocris Biosciences), (S)-3,5-dihydroxyphenylglycine (DHPG, mGluR1/5 agonist 10 μM, Tocris Biosciences) and (2R,4R)-amino-2,4-pyrrolidinedicarboxylic acid (APDC, mGluR2/3 agonist, 0.3 μM, Sigma Aldrich) were dissolved in 0.1% DMSO (except for DHPG dissolved in water) and added 2 h (except for DHPG, 1 h) before ACP (8 nM) or BCL (400 nM) to rat primary neuronal cells.

To treat the animals, compounds were administered *per os* twice daily at 8:00 am and 6:00 pm in a volume of 5 mL Kg^−1^ (mice) or 10 mL Kg^−1^ (rats). Aβ_25–35_ or Sc.Aβ was injected once for each experiment and treatment started 24 h before Aβ_25–35_ or Sc.Aβ or injection and lasted until the end of behavioural tests (see behavioural analyses below and Supplementary Table 2). For *h*APP_SL_ mice, treatment was started at 8 months of age and lasted 3–4 weeks until the end of functional tests (Supplementary Table 2). All behavioural tests were performed 2 h after treatment.

### Cell cultures

Cultures, Aβ_1–42_ intoxications and treatments were validated and performed at Neuronexperts laboratories (Marseille, France). Rat primary cortical and hippocampal neurons were cultured as described previously^21^. Briefly, 3 pregnant Wistar female rats of 15 days gestation for the use of cortical neurons or 17 days gestation for hippocampal neurons were killed by cervical dislocation and cortices of foetuses were dissociated. Cells were then mechanically dissociated with trypsin 0.05% (Pan Biotech, Aidenbach, Germany) and suspended after washing in a defined culture medium consisting of Neurobasal medium supplemented with 2% B27 (Invitrogen, Cergy Pontoise, France), 2 mM L-glutamine (Pan Biotech), 2% of Penicillin/Streptomycin (PS) and 10 ng mL^−1^ of brain-derived neurotrophic factor (BDNF, Pan Biotech). Cells were seeded in 96 well-plates pre-coated with poly-L-lysine (Greiner, Courtaboeuf, France) and were cultured at +37°C in a humidified air (95%)/CO_2_ (5%) atmosphere. After 11–13 days of culture of cortical neurons, cells were pre-treated for 1 h with ACP, BCL or combination (ABC) and then intoxicated with the appropriate concentration of Aβ_1–42_ during variable incubation times depending on the experiment (Supplementary Table 1). These different incubation times and concentrations were optimised to elicit only the toxic effects specific to the particular assay performed^21^. Seven different experiments with 18 replicates per experiment were performed and analysed. Hippocampal neurons were cultured during 18 days, then pre-treated for 48 h with ACP, BCL or ABC and finally intoxicated with Aβ_1–42_ during 48 h. Three different cultures with 6 replicates each were performed and analysed.

Mycoplasma-free Human Brain Microvascular Endothelial Cells (HBMEC, ref ACBRI 376, Cells System, Illinois, USA) were rapidly thawed at +37°C and immediately suspended in 9 mL Dulbecco's modified Eagle's medium (DMEM; Pan Biotech) containing 10% of foetal calf serum (FCS; Invitrogen). Cell suspensions were centrifuged at 180 *g* for 10 min at +4°C and pellets were suspended in CSC serum-free medium supplemented with 1.6% of Serum-free RocketFuel (Cell System), 2% of PS (Pan Biotech) and were seeded at the density of 20,000 cells per well in 96 well-plates (Matrigel Layer Biocoat Angiogenesis System, BD Biosciences, France) in a final volume of 100 μL (Supplementary Table 1). 3 different experiments with 18 replicates per experiment were performed and analysed.

### *In vitro* endpoint measurements

Before performing experiments with our candidate drugs, we validated experimental conditions with BDNF (50 ng/mL) for neuronal cultures or VEGF (10 nM) for endothelial cultures as positive controls to check and confirm the restoration and the validity of each endpoint after Aβ_1–42_ intoxication.

#### Neuroprotection

24 h after Aβ_1–42_ intoxication (Supplementary Table 1), culture supernatants were analysed with Cytotoxicity Detection Kit (LDH, Roche Applied Science, Meylan, France), a colorimetric assay based on the measurement of lactate dehydrogenase (LDH) released from the cytosol of dying cells, following the manufacturer's instructions.

#### Methionine sulfoxide (MetO), cytochrome c (Cyto c), Caspase 3, phosphorylated Tau (pTau), PSD95 and Synaptophysin

After Aβ_1–42_ intoxication (Supplementary Table 1), cells were fixed, permeabilised, and non-specific sites were blocked with a solution of phosphate buffered saline (PBS; PanBiotech) containing 0.1% of saponin (Sigma) and 1% FCS. Then, cells were incubated with primary antibody, co-stained with the other primary antibody, and revealed with secondary antibodies (Supplementary Table 3). Nuclei were counter-stained with Hoechst (Sigma). 10 to 40 pictures per well were taken in each experiment using the InCell Analyzer™ 1000 (GE Healthcare, France). Analysis was done using Developer software (GE Healthcare) assessing the overlap between MAP2 on one side, and MetO, Cyto c, Caspase 3 or pTau staining on the other side. PSD95 and Synaptophysin total surface overlap was quantified (in μm^2^) and computed for each condition. Results were expressed as the number of overlapping stained cells per field and reported as a percentage of vehicle control.

#### Glutamate release

After 4 h of Aβ_1–42_ intoxication (Supplementary Table 1), cell media supernatants were analysed with Amplex Red Glutamic Acid assay kit (Invitrogen) according to the manufacturer's instructions.

#### Capillary network quantification

2 pictures with a 4× lens were taken per well using InCell Analyzer™ 1000 in light transmission. Analysis of the tube formation was done using Developer software and the total length of capillary network was computed.

### Behavioural analyses

Animals were tested in a random and blind manner. Treatment groups were equally represented in each cage. Mice were habituated 30 min to 2 h before each experiment. Acute Aβ_25–35_ injection experiments (Supplementary Table 2) and readouts as well as novel object recognition studies were done and validated at Amylgen facilities (Montferrier-sur-Lez, France) for mice, and at Biotrial (Aβ_25–35_ experiments only) laboratories (Rennes, France) for rats. Transgenic mice studies were performed and validated at QPS facilities (Austria). Sample sizes used for behavioural analyses were also determined in each of these companies on the basis of previous experience on assay variability. Dead mice and mice that did not succeed preliminary control tests (i.e. very weak locomotor activity) were excluded from whole analyses.

#### Place learning in the Morris Water Maze^67^

Briefly, swimming was recorded using a Videotrack software (Viewpoint, Champagne-au-Mont-d'Or, France), with trajectories being analysed as latencies and distances. The software divides the pool into four quadrants. Training consisted in three swims per day for 6 days, performed between day 7 and 12 after Aβ_25–35_ injection, with 20 min between each swim. For *h*APP_SL_ mice, training consisted in 4 swims per day for 4 days. The latency, expressed as mean ± s.e.m., was calculated for each training day. A probe test was performed 24 h after the last swim, on day 13 after Aβ_25–35_ injection (retention phase). The platform was removed and each animal was allowed a free 60 s swim. The time spent in each quadrant was then determined. From Day 14 to 16 after Aβ_25–35_ injection, or after the training test performed in *h*APP_SL_ mice, animals were tested for spatial working memory. Working memory was specifically assayed by changing the platform location everyday (four swims per day during 3 days) and by using a training inter-trial time of 2 min. The swimming times to find platform of first (swim1), second (swim2), third (swim3) and fourth trial (swim4) of every day were calculated and averaged in Aβ_25–35_ mice experiments. In *h*APP_SL_ mice experiments, only data of day 2 trial were used.

#### Novel object recognition^34^

Briefly, 6 days after A*β*_25–35_ or Sc.A*β* peptide injection, mice were placed individually in a squared open-field and their locomotor activity was captured through an IR-sensitive camera and analysed using the Videotrack® software (Viewpoint) in terms of total distance travelled (cm). 24 h later (7 days after Aβ_25–35_ injection), two identical objects were placed at defined positions. Each mouse was placed in the open-field and the exploratory activity recorded during 10 min. The duration of contact with objects was analysed using the Nosetrack® protocol (Viewpoint). 24 h later (8 days after Aβ_25–35_ injection), the object in position #1 was replaced by a novel one differing in colour, shape and texture from the familiar object. Each mouse was placed again in the open-field and the exploratory activity recorded during 10 min. The preferential exploration index was calculated as the ratio of the duration of contacts with the object in position #1 over the total duration of contacts with both objects. For rats, 16 days after A*β* injection, rats were placed individually in a squared open-field and two trials spaced by 2 h interval were performed. During the first trial (acquisition trial), rats were placed in the arena containing 2 identical objects and time required by each animal to complete 15 s of object exploration were determined. For the second trial (testing trial), one of the objects presented in the first trial was replaced by an unknown object (novel object). Rats were placed back in the arena and exploration of each object was determined. For both trials, locomotor activity of rats was scored. Only animals having a minimal level of object exploration of 5 s during the testing trial were included in the study. The time spent in active exploration of the novel object and the familiar object (Delta Novel-Familiar) was measured.

#### Spontaneous alternation in the Y-maze^68^

7 days after Aβ_25–35_ or Sc.Aβ peptide injection, each mouse was placed at the end of one arm in a grey polyvinylchloride Y-maze. The series of arm entries, including possible returns into the same arm, was recorded visually. An alternation was defined as entries into all three arms on consecutive occasions. The number of the maximum alternations was the total number of arm entries minus two and the percentage of alternation was calculated as actual alternations/maximum alternations × 100. Measured parameters included the percentage of alternation (memory index) and total number of arm entries (exploration index).

#### Step through type passive avoidance^69^

Briefly, the apparatus consisted of two compartments with one illuminated with white polyvinylchloride walls and the other darkened with black polyvinylchloride walls and a grid floor for electrical shocks. A guillotine door separated each compartment. 8 days after Aβ_25–35_ or Sc.Aβ peptide injection, each mouse was placed into the white compartment. After 5 s, the door was raised. When the mouse entered the darkened compartment and placed all its paws on the grid floor, the door was closed and the foot shock (0.3 mA) delivered for 3 s. The latency spent to enter the dark compartment and the number of vocalisations were recorded. The number of vocalisations did not differ among groups, indicating that shock sensitivity was unaffected by the treatments (data not shown). The retention test was carried out 24 h after training (9 days after Aβ_25–35_ or Sc.Aβ peptide injection). Each mouse was placed again into the white compartment. After 5 s, the door was raised and the step-through latency (latency to enter the dark compartment) was recorded up to 300 s.

### Histology

Eight days after Aβ_25–35_ or Sc.Aβ injection, each mouse was anesthetised by intramuscular injection of 80 mg Kg^−1^ ketamine and 10 mg Kg^−1^ xylazine, and quickly perfused transcardially with 100 mL of saline solution followed by 100 mL of paraformaldehyde 4%. Brains were removed and kept for 24 h in the fixative solution at +4°C, then cut in coronal sections (20 μm) using a vibratome (Leica VT1000 S, Wetzlar, Germany). Sections were stained with 0.2% cresyl violet reagent (Sigma), dehydrated with graded ethanol, treated with toluene and mounted with Mountex medium (BDH Laboratory Supplies, Poole, Dorset, UK). Examination of the CA1 area was performed using a light microscope (Dialux 22, Leitz, Wetzlar, Germany), slices being digitalised through a CCD camera (Sony XC-77CE, Sony, Paris, France) with the NIH Image version 1.63 software. CA1 measurement and pyramidal cell count have been processed by ImageJ (NIH). Data were calculated as average of 8 slices and expressed as number of CA1 pyramidal cells per millimetre for each group.

### Blood-brain barrier (BBB) permeability

Seven days after Aβ_25–35_ or Sc.Aβ peptide injection, Evans Blue (EB) dye (2% in saline, 4 mL Kg^−1^) was injected i.p. 3 h prior to the transcardiac perfusion. Mice were anesthetised i.p. with 200 μL of pre-mix 80 mg Kg^−1^ ketamine and 10 mg Kg^−1^ xylazine, then perfused transcardially with 250 mL of saline solution. After decapitation, the brain was removed, dissected and weighed for quantitative measurement of EB extravasation. Samples were homogenized in PBS and mixed by vortexing after the addition of 60% trichloroacetic acid to precipitate the protein. Samples were cooled at +4°C, and then centrifuged 30 min at 10,000 *g* at +4°C. The supernatant was measured at 610 nm for absorbance of EB using a spectrophotometer. EB quantification was expressed as pg per mg of brain tissue.

#### Biochemical analyses

Seven (synaptophysin), 8 (GFAP), or 9 (Aβ_1–42_, cPARP, BDNF, IL-1β, TNFα) days after Aβ_25–35_ or Sc.Aβ injection, hippocampi were dissected out and rinsed in ice-cold PBS to remove excess blood, and weighed before nitrogen freezing and −80°C storage. Tissues were cut into small pieces, homogenised, sonicated and centrifuged. The supernatants were assayed immediately by ELISA for GFAP, synaptophysin, IL-1β (USCN, China), TNFα (Thermo Scientific, France), BDNF (Promega, France), cPARP (Cell Signalling, France) and Aβ_1–42_ (Euromedex, France) following manufacturer's recommendations. All samples were assayed in duplicate and only samples for which the CV was <25% between replicates were included for analyses. Results were expressed in pg protein per mg of brain tissue, or as % of Sc. control. Protein concentration was determined in brain homogenates with the BCA protein assay kit (Pierce Perbio Science, France).

### Lipid peroxidation

Briefly, mice were killed by decapitation and hippocampi were rapidly isolated, weighed, and kept in liquid nitrogen until assayed. After thawing, brains were homogenised in cold methanol (1:10, w/v), centrifuged at 1,000 *g* during 5 min at room temperature and supernatants collected. Homogenate was added to a solution containing 1 mM FeSO_4_, 0.25 M H_2_SO_4_, 1 mM xylenol orange, and incubated for 30 min at room temperature. Absorbance was measured at 580 nm (A_580_1), and 10 μL of 1 mM cumene hydroperoxide (CHP) was added to the sample and incubated for 30 min at room temperature to determine the maximal oxidation level. Absorbance was measured at 580 nm (A_580_2). The level of lipid peroxidation was determined as CHP equivalents according to: CHP equivalent = A_580_1/A_580_2 × (CHP (nmol)) × dilution, and expressed as CHP equivalents per mg tissue.

### Statistical analyses

Statistical tests were two-tailed, and conducted at a 5% significance level. Sample sizes used for behavioural analyses were defined on the basis on previous experience on assay variability. Data distribution and within-group variation were preliminary assessed in order to guide our methodological choices. We applied an Analysis of Variance (ANOVA) with Dunnett's test for comparison of more than one experimental group against a reference. A *t*-test was also used as a supportive technique. For repeated measures data (MWM acquisition and working memory tests in Figures 2a,b,f,g) we applied a mixed ANOVA with Dunnett's test, including fixed effect terms for treatment, time and the treatment by time interaction, and a random effect term for animals. Treatment effect was assessed at each time point and for combined time points (global effect). Group comparisons were performed with Prism (http://www.graphpad.com/scientific-software/prism).

Drug combination analyses with calculation of Combination Indexes (CI) and isobolograms^70^ were performed with *R* (http://cran.r-project.org). Briefly, CI compares dosages for a given combination to those expected to obtain the same combination effect under a simple additive assumption, and offers a quantitative definition for additive effect (CI = 1), synergism (CI < 1), and antagonism (CI > 1) in drug combinations. Isobologram is a popular graph introduced by Loewe^71^ constructed for a given effect level on a coordinate system composed of the individual drug doses (Supplementary Fig. 1c). In particular, doses of drug A and drug B (each alone) that give this effect are plotted as axial points. The line connecting these points represents dose pairs that will produce this effect in a simply additive combination. This line of additivity allows a comparison with the actual dose pair that produces this effect level experimentally, and is employed to distinguish additive from synergistic (below the line) and antagonistic (over the line) combinations.

## Supplementary Material

Supplementary Information Supplementary Information
