# Supplementary material for: Combining two repurposed drugs as a promising approach for Alzheimer's disease therapy
Source: Sci Rep. 2015 Jan 8;5:7608. doi: 10.1038/srep07608 (PMC5378993; doi:10.1038/srep07608)
Supplement: Supplementary Information [file srep07608-s1.pdf]

Supplementary Information

Combining two repurposed drugs as a promising approach for Alzheimer's disease therapy

Ilya Chumakov[1], Serguei Nabirotchkin[1], Nathalie Cholet[1], Aude Milet[1], Aurélie Boucard[1], Damien Toulorge[1], Yannick Pereira[1], Esther Graudens[1], Sory Traoré[1], Julie Foucquier[1], Mickael Guedj[1], Emmanuel Vial[1], Noëlle Callizot[2], Rémy Steinschneider[3], Tangui Maurice[4,5], Viviane Bertrand[1], Catherine Scart-Grès[1], Rodolphe Hajj[1] & Daniel Cohen[1*]

[1]Pharnext, 11 rue des Peupliers, 92130 Issy-Les-Moulineaux, France.

[2]Neuro/Sys, 410 CD 60, 13120 Gardanne, France.

[3]Neuronexperts, 51 bd Pierre Dramard, 13916 Marseille, France.

[4]Université de Montpellier 2, 34095 Montpellier, France; Inserm, U710, 34095 Montpellier, France; EPHE, 75017 Paris, France.

[5]Amylgen, 2196 bd de la Lironde, 34980 Montferrier-sur-Lez, France.

* Correspondence should be addressed to D.C (dcohen@pharnext.com)

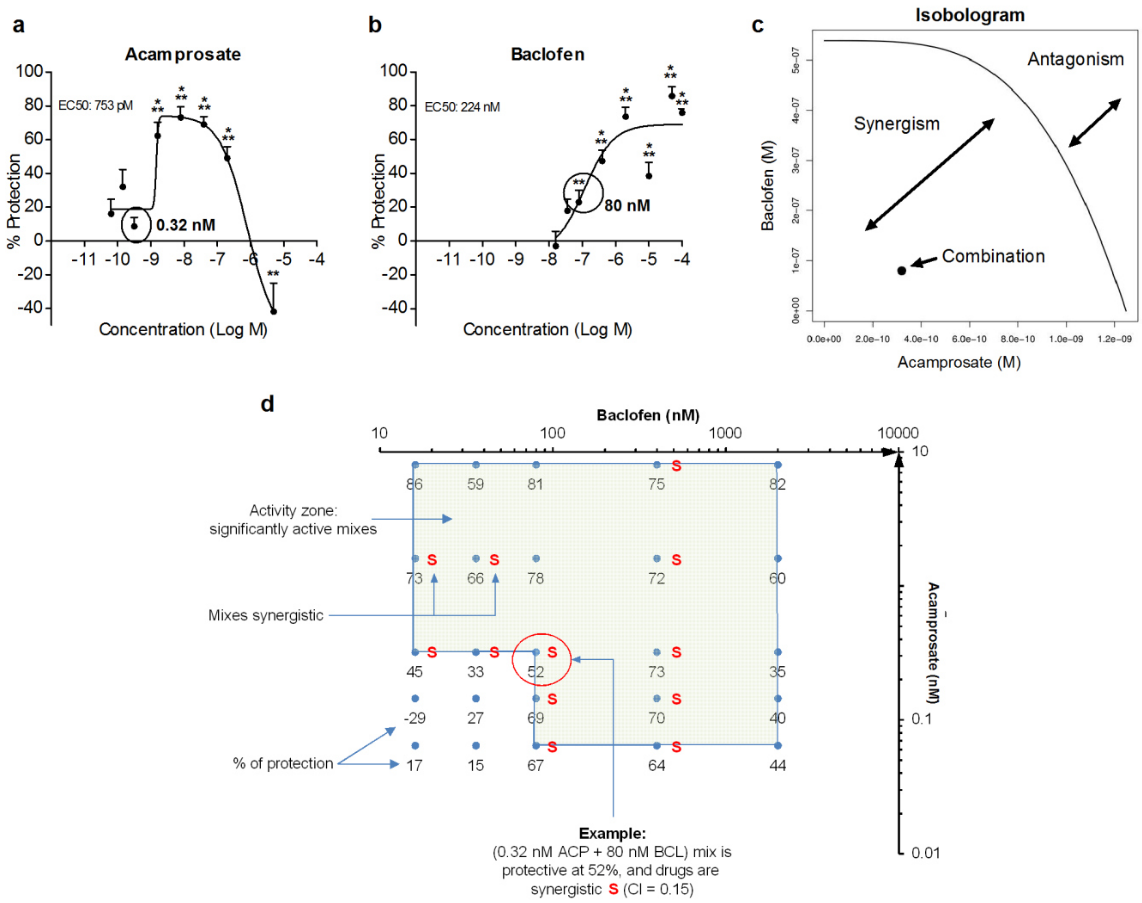

Supplementary Figure 1 ACP and BCL combination is synergistic. Example of synergism between ACP and BCL. Data are derived from a combined analysis of 7 different experiments with 18 replicates per experiment. Data were normalized to the non-treated control (100%) and $A\beta_{1\text{-}42}$-intoxicated cells (0%). (**a,b**) Dose-response of ACP and BCL respectively, assessed in the rat primary cortical neuronal model of neuroprotection. Sub-active doses of 0.32 nM of ACP and 80 nM of BCL (i.e. doses that are inactive in the cellular model or weakly active) were combined and used to treat Aβ-intoxicated neuronal cells. Panel (**c**) shows the isobologram of the combined drugs from (**a**) and (**b**), with a Combination Index (CI) of 0.15 and a positive protective effect of 52%. The effect of this

synergistic combination was superior to the sum of the effects of its single drugs (see **Fig. 1c**). Values are mean ± s.e.m. $^{**}P < 0.01$, $^{***}P < 0.001$ versus Aβ, ANOVA with Dunnett's test. (**d**) A dose effect study of ACP and BCL combination (ABC) was performed to check for synergism for a wide range of concentrations and drugs ratios. Several concentrations of ABC were active (activity zone), and several combinations of ACP and BCL were synergistic (**S** in red). Numbers under the dots represent the percentage of protection.

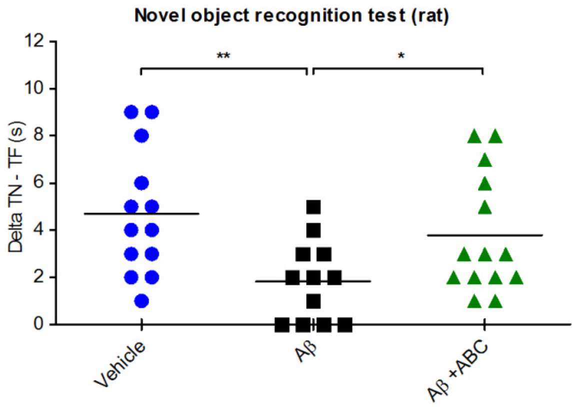

Supplementary Figure 2 ABC is active in the novel object recognition (NOR) task performed in male Sprague Dawley rats. 15 nmol of oligomeric $A\beta_{25\text{-}35}$ were injected intracerebroventricularly in each rat. Times spent in exploring the familiar (TF) and the novel (TN) objects were measured, and the delta TN-TF was computed for each animal. ABC positively alleviated cognitive deficit induced by $A\beta_{25\text{-}35}$ in rats. Values are mean ± s.e.m. $^{*}P < 0.05$, $^{**}P < 0.01$ versus Aβ; *t*-test (n = 12 - 14 animals per group). ABC was composed of ACP 0.1 mg Kg^{-1} and BCL 1.5 mg Kg^{-1}, which is the rat equivalent dose of ACP 0.2 mg Kg^{-1} and BCL 3 mg Kg^{-1} in mice based on body surface.

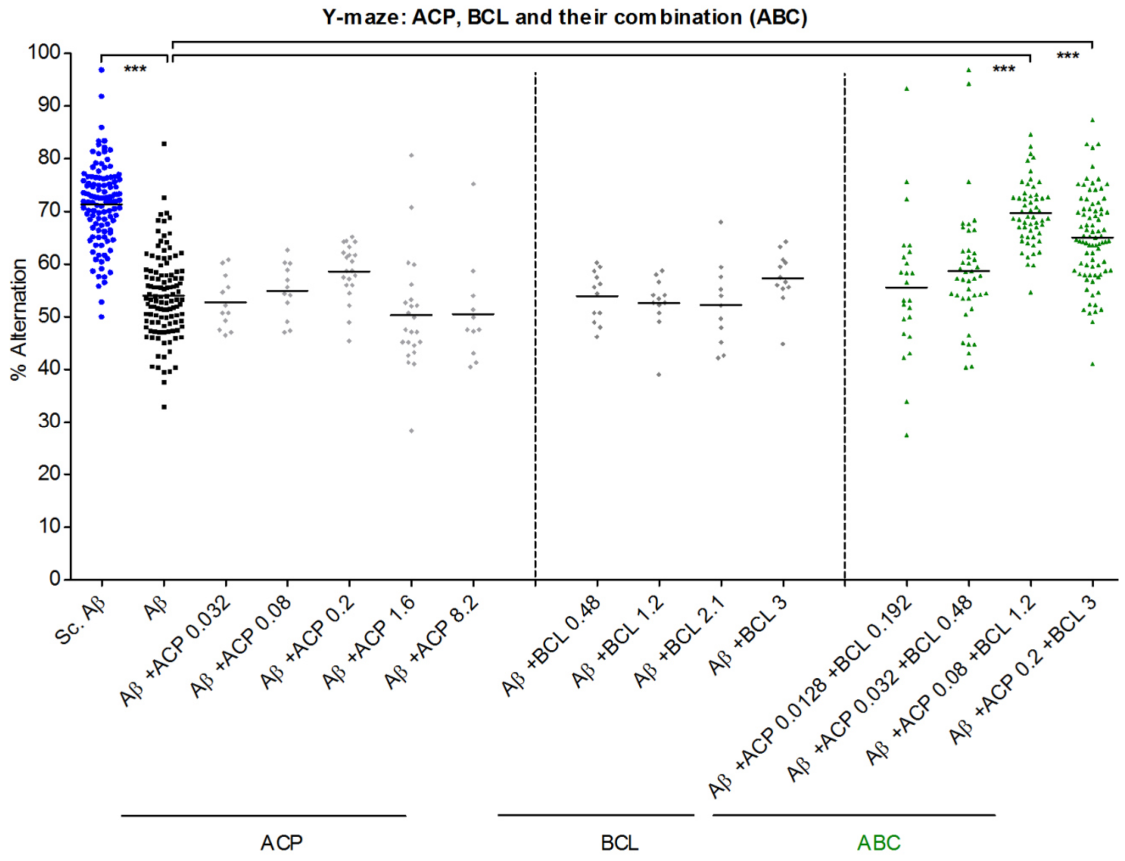

Supplementary Figure 3 ABC synergistically rescues cognitive deficit in Aβ$_{25-35}$ male Swiss mice in the Y-maze test. Dose-response effect of ACP, BCL and their combination (ABC) in the spatial working memory Y-maze test performed in mice. For its ease of use, this test was used to screen ACP and BCL concentrations. Each mouse was injected intracerebroventricularly with 9 nmol of oligomeric Aβ$_{25-35}$. Data are derived from a combined analysis performed on 10 independent experiments in mice (*n* = 12 per group per experiment), where each experiment was normalized to the global mean, over all the experiments, of the Aβ group and the Sc.Aβ group. It is to be noted that some doses such as ACP 0.2 mg Kg^{-1} and BCL 3 mg Kg^{-1} show an activity much weaker than their mix. ****P* < 0.001 versus Aβ; ANOVA with Dunnett's test. Sc.: scrambled.

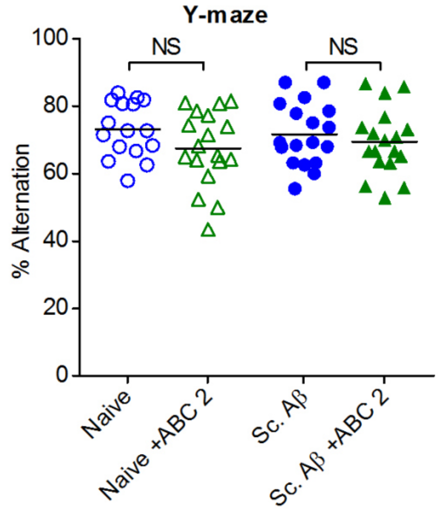

Supplementary Figure 4 ABC does not affect cognition of control mice. Normal naive, or intracerebroventricularly Sc.A$\beta_{25\text{-}35}$-injected Swiss male mice were treated with ABC and assayed in the Y-maze test (n = 18 per group). No significant difference was observed between controls and ABC-treated animals (t-test). ABC did not modify the cognition of treated mice in the absence of a pathological action of A$\beta_{25\text{-}35}$. ABC 2 was composed of ACP 0.2 mg Kg^{-1} + BCL 3 mg Kg^{-1}. NS: not significant; Sc.: scrambled.

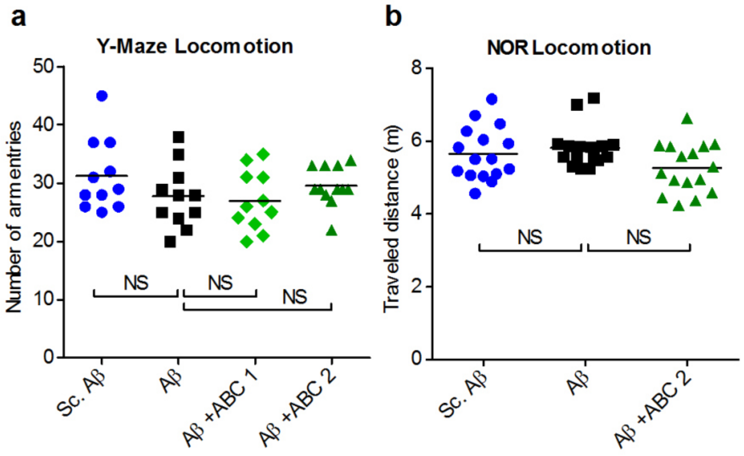

Supplementary Figure 5 Locomotor activity of Swiss male mice is not significantly affected in the Y-maze and NOR tests. (**a**) Mice behaved normally in the Y-maze test and did not exhibit any significant hyperactivity due to Aβ injection or to ABC treatment, as assessed by the number of arm entries (*n* = 12 animals per group). (**b**) Mice behaved normally in the NOR task and did not exhibit any significant hyperactivity due to Aβ injection or to ABC treatment as assessed by the total distance travelled (*n* = 16 animals per group). ANOVA with Dunnett's test. ABC 1: ACP 0.08 mg Kg^{-1} + BCL 1.2 mg Kg^{-1}; ABC 2: ACP 0.2 mg Kg^{-1} + BCL 3 mg Kg^{-1}. Sc.: scrambled.

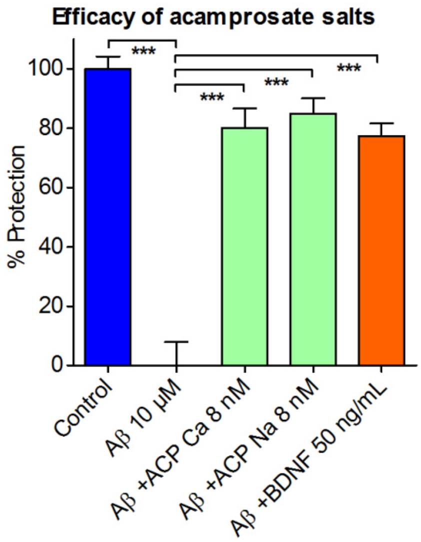

Supplementary Figure 6 ACP is the active molecule in the ACP calcium formulation. Data were normalized to the non-treated control (100%) and $A\beta_{1\text{-}42}$-intoxicated cells (0%). The panel describes ACP Ca and ACP Na neuroprotective effect in the rat primary cortical neuronal model. Protection was equivalent with both ACP forms highlighting the effect of ACP molecule and minimizing that of its salt. BDNF was used as positive control. Values are mean ± s.e.m. ***$P < 0.001$ versus Aβ; ANOVA with Dunnett's test. ACP Ca: Calcium salt of acamprosate; ACP Na: Sodium salt of acamprosate.

Supplementary Table 1 Characteristics of *in vitro* experiments.

			$A\beta_{1-42}$			
**Assay**	**Days of culture**	**Seeding density (cells/well)**	**Concentration**	**Beginning of intoxication**	**Intoxication time (h)**	**Measured endpoint**
**LDH**[†]	12	30,000[‡]	10 µM	D11	24	LDH release
**MetO**	11	15,000[‡]	1.25 µM	D11	4	Number of stained neurons*
**Cyto c**	11	30,000[‡]	1.25 µM	D11	4	Number of stained neurons*
**Caspase 3**	11	30,000[‡]	10 µM	D11	24	Number of stained neurons*
**Tau phosphorylation**	12	30,000[‡]	2.5 µM	D11	16	Number of stained neurons*
**PSD95/Synaptophysin**	22	20,000[¤]	0.3 µM	D20	48	Surface overlap between markers
**Glutamate release**	13	30,000[‡]	2.5 µM	D12	4	L-glutamic acid
**Capillary Network**	Passage 10	20,000[#]	2.5 µM	2 h	18 (2 h after HBMEC seeding on Matrigel)	Total length of capillary network

[‡]Cortical neurons.

[†]Lactate dehydrogenase.

*Staining overlapping with MAP2 staining.

[¤]Hippocampal neurons.

[#]HBMEC.

Supplementary Table 2 Characteristics of *in vivo* experiments.

Model	Assay	Measured parameters	Start of ABC treatment (*b.i.d.*)*	Injections†	Days of test	Total duration of treatment (Days)
$A\beta_{25–35}$	Morris Water Maze	Acquisition (escape latency)	D-1	$A\beta_{25–35}$ D0	D7-D12	14
$A\beta_{25–35}$	Morris Water Maze	Working memory (escape latency)	D-1	$A\beta_{25–35}$ D0	D14-D16	18
$A\beta_{25–35}$	Novel Object Recognition mice	Exploration (% time of exploring novel object)	D-1	$A\beta_{25–35}$ D0	D6-D8	10
$A\beta_{25–35}$	Novel Object Recognition Rats	Explorations (time of exploring novel object)	D-1	$A\beta_{25–35}$ D0	D15-D16	18
$A\beta_{25–35}$	Y-Maze	Alternations (%)	D-1	$A\beta_{25–35}$ D0	D7	9
$A\beta_{25–35}$	Passive avoidance	Retention (step through latency)	D-1	$A\beta_{25–35}$ D0	D8-D9	11
$h\mathrm{APP}_{SL}$	Morris Water Maze	Acquisition (escape latency)	Month 8 of age (D0)	NA‡	D17-D20	20
$h\mathrm{APP}_{SL}$	Morris Water Maze	Working memory (escape latency)	Month 8 of age (D0)	NA	D22-D24	24

**b.i.d*: *bis in die.*

†Injected once. Mice were also treated with ABC *b.i.d.*

‡Not applicable.

Supplementary Table 3 Antibodies used for *in vitro* endpoints.

Antibodies	Dilution	Distributor	Catalogue reference / Clone
Primary Antibodies			
Mouse monoclonal Ab* to MAP2	1/400	Sigma Aldrich, France	M4403 / HM-2
Rabbit polyclonal Ab to MetO	1/100	Euromedex, France	MS01
Rabbit polyclonal Ab to Cyto c	1/100	Abcam, France	ab90529
Rabbit polyclonal Ab to Caspase 3	1/500	Sigma, France	C8487
Mouse monoclonal Ab to PSD95	1/100	Abcam, France	ab2723
Rabbit polyclonal Ab to Synaptophysin	1/100	Abcam, France	ab7837
Chicken polyclonal Ab to MAP2	1/400	Abcam, France	ab5392
Mouse monoclonal Ab to PHF[†]-Tau, clone AT100	1/100	Thermo Scientific, France	MN1060 / AT100
Secondary Antibodies			
Alexa Fluor 488 goat Ab to mouse IgG			A11001
Alexa Fluor 568 goat Ab to rabbit IgG	1/400	Invitrogen, France	A11011
Alexa Fluor 568 goat Ab to chicken IgG			A11041

*Antibody

[†]PHF: Pair Helical Filament.
